# Investigation on Surface Integrity of Rapidly Solidified Aluminum RSA 905 by Magnetic Field-Assisted Finishing

**DOI:** 10.3390/mi9040146

**Published:** 2018-03-25

**Authors:** Jiang Guo, Hao Wang, Min Hao Goh, Kui Liu

**Affiliations:** 1 Key Laboratory for Precision and Non-traditional Machining Technology of Ministry of Education, Dalian University of Technology, Dalian 116024, China; 2 Department of Mechanical Engineering, Faculty of Engineering, National University of Singapore, EA-02-05, 9 Engineering Drive 1, Singapore 117575, Singapore; mpewhao@nus.edu.sg; 3 Singapore Institute of Manufacturing Technology, 73 Nanyang Drive, Singapore 637662, Singapore; gohmh@simtech.a-star.edu.sg (M.H.G.); kliu@simtech.a-star.edu.sg (K.L.)

**Keywords:** rapidly solidified aluminum, magnetic field-assisted finishing (MFAF), material removal, surface integrity, residual stress, tribology

## Abstract

RSA 905, a rapidly solidified aluminum alloy, has been widely utilized in optical, automotive, and aerospace industries owing to its superior mechanical properties such as hardness and strength compared to conventional aluminum alloys. However, the surface finishing of RSA 905 to achieve submicron surface roughness is quite challenging and was rarely addressed. This paper presents an experimental and analytical study on magnetic field-assisted finishing (MFAF) of RSA 905 through a systematic investigation on surface integrity in relation to the MFAF process parameters. The effect of abrasive and polishing speed conditions on material removal and surface roughness was quantitatively investigated. The surface and subsurface quality were evaluated by optical microscope and scanning electron microscope (SEM) observations, residual stress measurement, surface microhardness and tribology test. The results show that relatively high material removal and low surface roughness were obtained under conditions using the SiC abrasive with a grit size of 12 µm at polishing speed of 400 rpm or using the Al_2_O_3_ abrasive with a grit size of 5 µm at polishing speed of 800 rpm. Heat melt layer caused by wire electrical discharge machining (EDM) during the sample preparation was removed by MFAF without inducing new subsurface damage. The MFAF process also helps release the surface residual stress and improve the tribological performance although the surface microhardness was slightly reduced.

## 1. Introduction

Material property and function are intimately dependent on microstructure such that ultra-fine grained (UFG) materials are in general harder, tougher and more wear-resistant compared to their coarse-grained versions [1]. Various material treatment methods have been employed to improve the material properties through modifying the microstructures. Large strain or severe plastic deformation processes such as equal channel angular pressing or extrusion (ECAP, ECAE) [2,3,4,5,6] have been broadly used to refine the microstructure of precipitation-strengthened and age-hardening alloys, and produce thermally stable UFG aluminum alloys owing to the precipitate phase-induced thermal stability by dislocation and grain boundary pinning [7]. Towards its applications, the machinability of UFG materials such as UFG copper and steel has been experimentally assessed [8,9]. Rapid solidification technology (RST) has also played a key role in enhancing the mechanical behavior of metallic systems through producing ultra-fine microstructures, especially for lightweight metal alloys since the 1980s [10].

**Figure 1:**
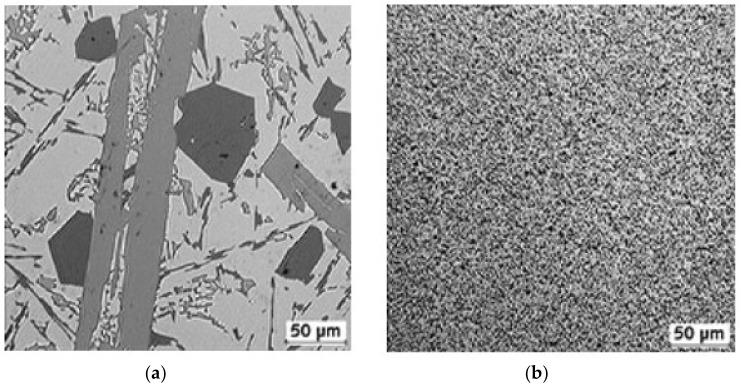
Microstructures of (**a**) conventional aluminum and (**b**) rapidly solidified aluminum [11].

Rapidly solidified aluminum, owing to its superior mechanical properties of hardness and strength over conventional light-weight aluminum alloys, has a wide application in optical, automotive, aerospace and even sports industries. As a superior alternative material for injection mold inserts, RSA 905 with an average grain size of 1–2 µm has been developed by the rapid solidification process. As shown in Figure 1, the grain size of rapidly solidified aluminum is much smaller than that of the conventional aluminum alloy. In terms of physical and mechanical properties, RSA 905 contains more constituent elements such as Fe, Ni and Cu, and has the same density with the conventional aluminum alloy but about two times higher tensile strength, yield strength, and hardness [11].

To explore the optical applications, diamond turning has been employed to finish the mirror surface on RSA 905 by a group of researchers. Abou-El-Hossein et al. investigated diamond tool wear in machining of RSA 905 and discussed a special case with the presence of Fe and Ni particles. The effect of machining parameters on surface roughness was briefly discussed based on the surface texture parameter Ra (arithmetic average surface roughness) [12,13]. However, ultraprecision optical surface imposes more stringent requirements on the surface finish which can no longer be fully characterized by the Ra value. Such high-quality optical surfaces should be free of microcracks, machining marks, or porosity. Microcracks could be readily avoided in machining ductile materials such as aluminum alloys; however, machining marks are the intrinsic feature or lay pattern or signature of a certain machining process and the porosity due to the granular grain structure of RSA 905 is typically observed on the machined surface which is independent of the Ra value. In this regard, an appealing value of roughness does not guarantee superior functions of the optical surfaces manufactured on RSA 905. Although several research works reported the polishing of RSA 905 using magnetorheological finishing (MRF) [14] and chemical mechanical polishing [15], these prior works targeted an improvement in the Ra value but were not dedicated to promoting the surface integrity in terms of the granular defects induced by the mechanical material removal process. Besides, for most of non-optical applications in automotive, aerospace and sports industries, nanometer level of surface roughness is not required and micrometer level of surface roughness Ra is usually acceptable. Thus, in this paper, a magnetic field assisted finishing method is applied to achieve the surface quality of rapidly solidified aluminum RSA 905 and improve material removal efficiency which cannot be directly obtained by diamond turning.

Magnetic field-assisted finishing (MFAF), as one of the widely used polishing technologies, seems to be more suitable for complex geometries as magnetic abrasives are employed as the flexible tool [16,17,18]. The utilization of MFAF allows very complex geometry of the workpiece to be accessed. Compared with other polishing technologies such as bonnet polishing [19], Magnetorheological Finishing (MRF) [20], miniaturized vibrating tool polishing [21], conical pin-type and conical wheel-type tools polishing [22], and even fluid jet polishing [23], MFAF enables better geometry conformant capability for freeform and microstructured surfaces. It also exhibits good flexibility in controlling process parameters such as magnetic abrasive composition and polishing force, to reach target user requirements in work tolerances and surface conditions. Our preliminary work proved that MFAF process can achieve surface roughness less than 100 nm which not only meets industry requirements but also can be used to finish free-form or microstructured surfaces [24]. Since this finishing method is not associated with any tool wear, it is more efficient and flexible than diamond turning process to fabricate high-quality surfaces in a large format. This paper presents an experimental and analytical study on material removal characteristics of RSA 905 by MFAF, aiming to clarify the interrelation between surface/subsurface quality and process parameters. The effect of abrasive and polishing speed conditions on material removal and surface roughness was quantitatively investigated. The surface and subsurface quality were evaluated through optical microscope and scanning electron microscope (SEM) observations, residual stress measurement, surface microhardness and tribology test.

## 2. Experimental

### 2.1. Experimental Setup

**Figure 2:**
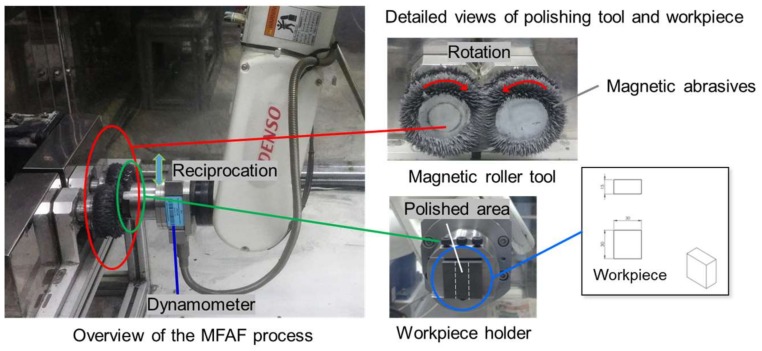
Experimental setup.

As shown in Figure 2, the experimental setup mainly consists of a dual magnet roller tool, a 6 degrees of freedom (DOF) robot arm, a dynamometer, as well as a workpiece mounting fixture. The dual magnetic roller design was adopted to generate the magnetic field. It was designed in a manner that exhibits differential magnetic flux densities at various target positions around the rollers, enabling a reforming mechanism of the magnetic abrasives at each revolution [25]. It was attached to 2 rotary motors that provide a controlled variable speed motion up to 1800 rpm. The magnetic abrasives accumulated between the two rollers were used as the polishing tool to remove material from workpiece surface. The use of the robot arm enables the use of a programmable work path to reach non-planar workpiece profiles. The detailed conditions used in the experiments are listed in Table 1. The workpieces were cut into a dimension of 30 mm × 30 mm × 15 mm by wire-cutting, followed by surface preparation for target initial conditions via wire electrical discharge machining (EDM) with an initial surface roughness Ra over 3.0 µm. The rotation speed of the dual magnetic roller tool was set to 400 rpm while the robot arm fed at a speed of 288 mm/min (40% of the maximum speed) vertically with a reciprocation distance of 60 mm. The gap between the roller and the workpiece was set to 1.5 mm. The center part of workpiece surface was polished. The force exerted by the magnetic abrasives on workpiece surface was about 20 N which was measured by the dynamometer.

Two kinds of abrasives, SiC and Al_2_O_3_, were adopted for the experiments. SiC and Al_2_O_3_ have the same weight percentage in the magnetic abrasives so there is a slight difference in volume. The size of iron powders was around 10 µm. For the type of SiC-based magnetic abrasives, the volume composition was taken at 30.9% Carbonyl Iron Powder (CIP) CM grade, 6.8% SiC powder, 54.2% lubricating fluid, and 8.1% machining oil. For the type of Al_2_O_3_-based magnetic abrasives, the volume composition was taken at 30.5% CIP CM grade, 8.3% Al_2_O_3_ powder, 53.2% lubricating fluid, and 8.0% machining oil. Ecocool, a type of lubricating fluid, is used in place of conventionally used water in the abrasive composition. It is used to reduce the fiction between magnetic abrasives and workpiece to prevent surface damage cause by friction-induced heating at higher tool speed experiment conditions [26,27]. During experiments, about 10 mL of lubricating fluid was replenished in the magnetic abrasives at fixed intervals of 5 min and the magnetic abrasives were replenished every 10 min. The material removal and surface roughness were measured using a contact type, stylus profilometer (Form Talysurf PGI 2540, Taylor Hobson, Leicester, UK) with a vertical resolution of 0.2 nm. The measurement settings such as cut-of-length and filtering conditions follow ISO standard. The stylus profilometer scanned across the polished area [26].

### 2.2. Design of Experiments

A full factorial experiment is designed to study the influences of different factors on the surface roughness and material removal rate in the MFAF process. The design of experiments is given in Table 2 where abrasive type, abrasive size, and polishing speed are considered at two levels, respectively. The polished surface roughness and material removal rate will be measured with 3 repetitions. The analysis of variance (ANOVA) will be further conducted to identify the significance and interactions of different process parameters and the optimal polishing conditions will be concluded for the MFAF process.

## 3. Results and Discussions

### 3.1. Surface Roughness, Morphology, and Material Removal

**Figure 3:**
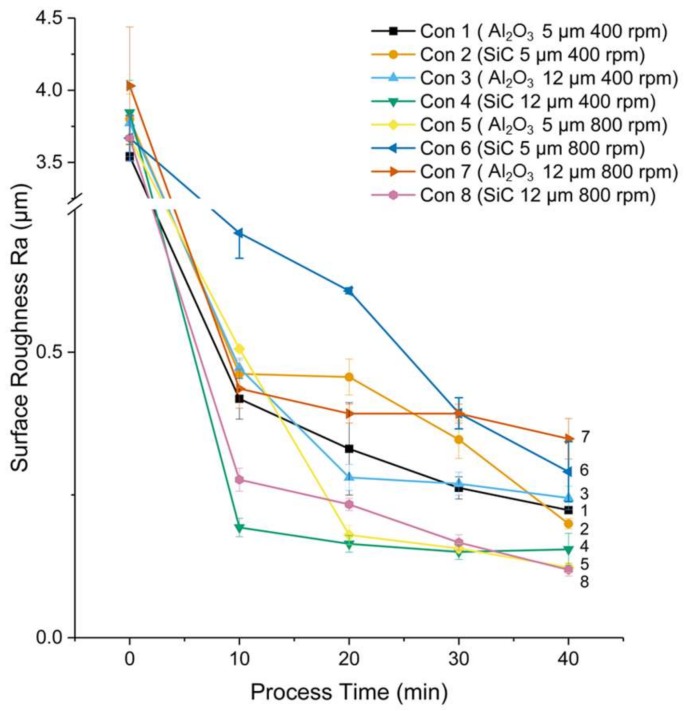
Change of surface roughness as a function of polishing time.

**Figure 4:**
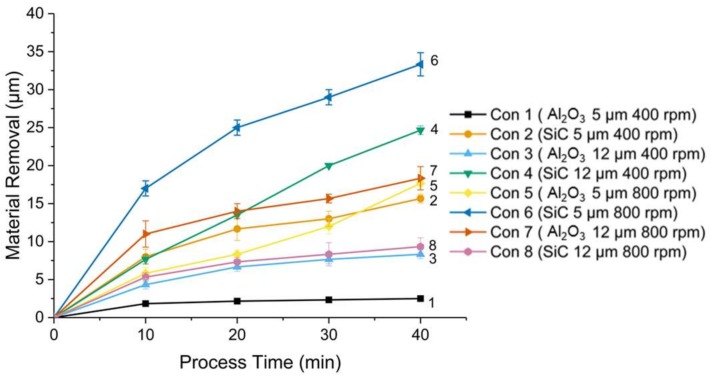
Change of material removal as a function of polishing time.

The effect of abrasive type and size on surface roughness, morphology, and material removal was evaluated. The trends of surface roughness and material removal change as a function of polishing time are shown in Figure 3 and Figure 4. Five data were taken for each test along the processing time for both surface roughness and material removal. As shown in Figure 3, the surface roughness dropped dramatically in the first 10 min, and it was reduced faster under the condition of test 4 than other types of abrasives. Then it gradually saturated and reached about 0.3 µm Ra after 40 min. polishing.

Material removal increased with the increment of polishing time (see Figure 4). At the polishing speed of 400 rpm, it is found that the type of SiC-based magnetic abrasives produced a higher material removal than that of Al_2_O_3_-based magnetic abrasives, and large particles contributed to a higher material removal. However, at the polishing speed of 800 rpm, due to the high rotation speed, large particles became susceptible to being scattered and dropped out from the abrasives. Due to such an experimental observation, the reduced volume of the SiC and Al_2_O_3_ particles in turn resulted in a lowered material removal. It should be noted that the material removal of “Con 1” increased a little bit but not stopped. Plowing or burnishing effect may happen but need to be further confirmed. Therefore, for the Al_2_O_3_-based magnetic abrasives, particle sizes of 5 µm and 12 µm achieved similar material removal after 40 min polishing. For the type of SiC-based magnetic abrasives, material removal by particle size of 12 µm was much lower than that by particle size of 5 µm. 

**Figure 5:**
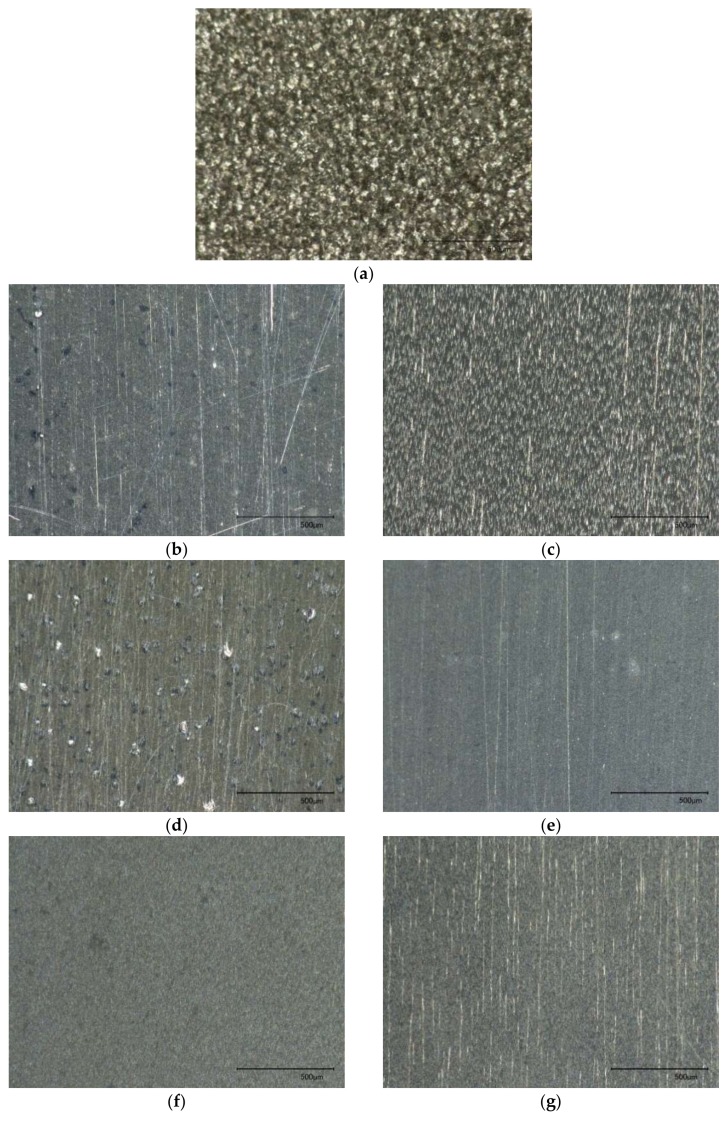

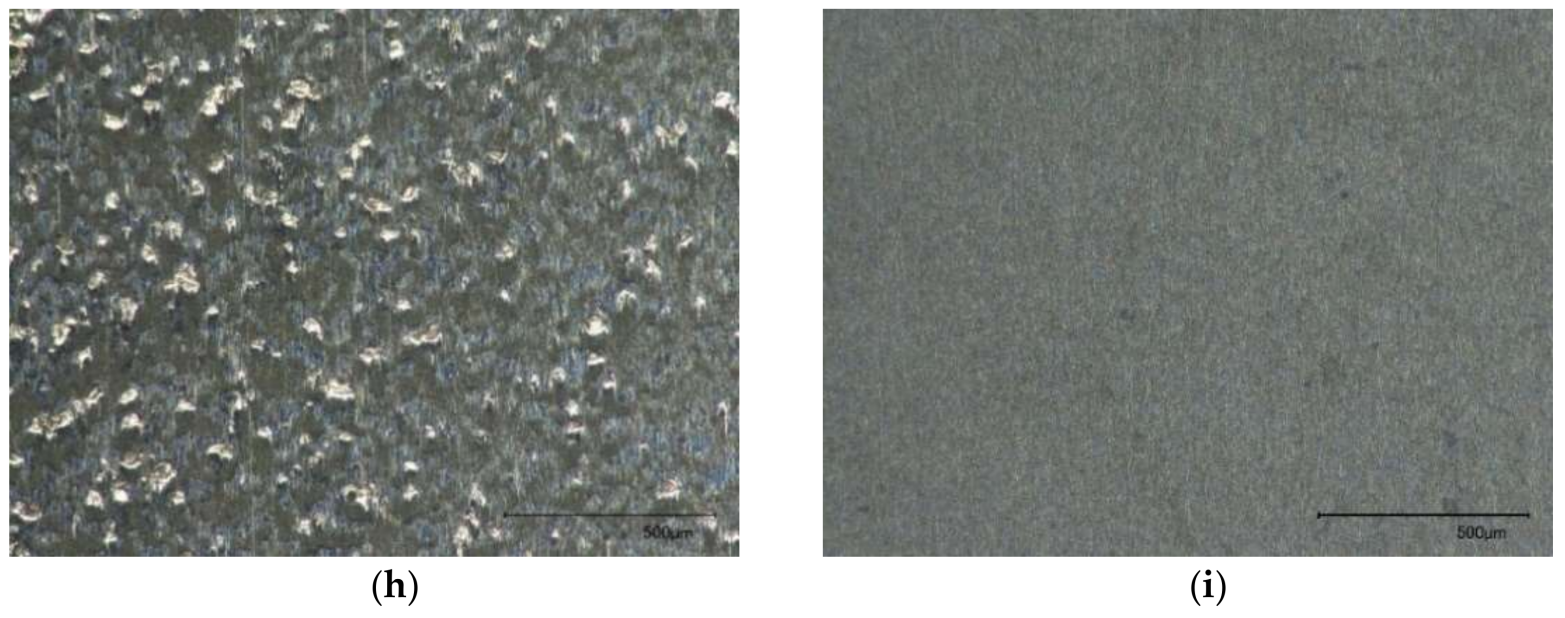
Surface morphologies of (**a**) unpolished surface as prepared by wire EDM and (**b**–**i**) polished surfaces by test conditions of 1–8. (The scale bar is 500 µm in length).

Although the surface roughness in all the 8 tests achieved a similar level, the surface morphologies which were measured by an optical microscope were significantly different. As shown in Figure 5, the workpiece surface polished by the SiC-based magnetic abrasives featured deep and long scratches while the Al_2_O_3_-based magnetic abrasives were associated with long scratches and small swells. It was further identified that the small swells were caused by the adhesive wear with spread material.

From the statistical analysis of ANOVA, according to P Value in Table 3, it can be concluded from that at the 0.05 level, abrasive type is the significant factor for the polished surface roughness Ra while the population means of abrasive size and polishing speed are not significantly different. The significant contribution of the interaction between different polishing parameters can also be identified such as “abrasive type * abrasive size” and “abrasive type * abrasive size * speed”. Thus, abrasive type is the critical factor for the developed MFAF process in terms of surface roughness Ra. Similarly, the significant factors for different MFAF parameters at the 0.05 level are listed in Table 4. All the parameters are dominant for the entire MFAF process regarding material removal.

### 3.2. Microstructure and Cross Sectional Observation

**Figure 6:**
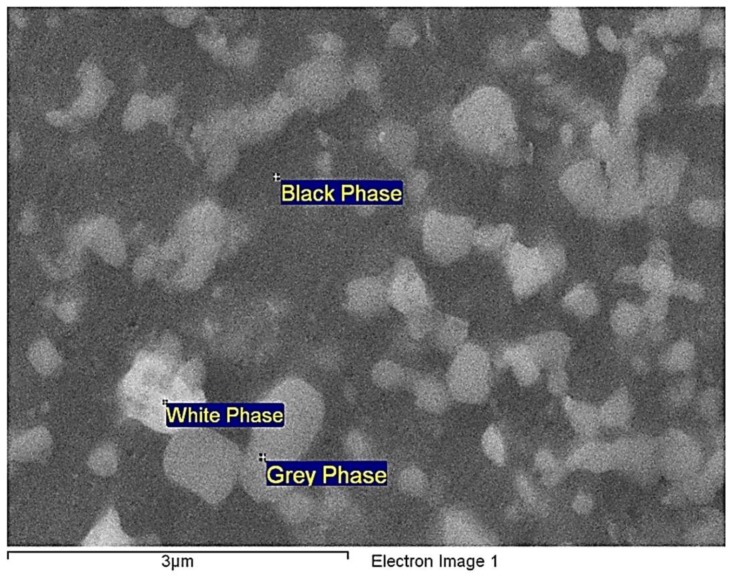
Microstructure and phases of RSA 905.

To observe the microstructure and subsurface quality of RSA 905, samples were prepared for cross sectional observation. The samples were cut to smaller pieces by wire EDM. The cross-sectional samples were then cold mounted with slow cure epoxy, followed by mechanical grinding and polishing up to 0.05 µm colloidal silica suspension. Then, an Ultra Plus Field-Emission Scanning Electron Microscope (FESEM) from Carl Zeiss was employed to characterize both the bulk’s microstructures and cross-sectional surfaces after the EDM and MFAF processes. For cross sections, secondary electron images using 10 kV accelerating voltage were taken near the transition zone where EDM and MFAF were performed. As shown in Figure 6, the microstructures of RSA 905 consist of ultra-fine grains of 100 nm to 1000 nm in size. From the secondary electron images, distinct white and grey phases were observed in the matrix of black phase.

**Figure 7:**
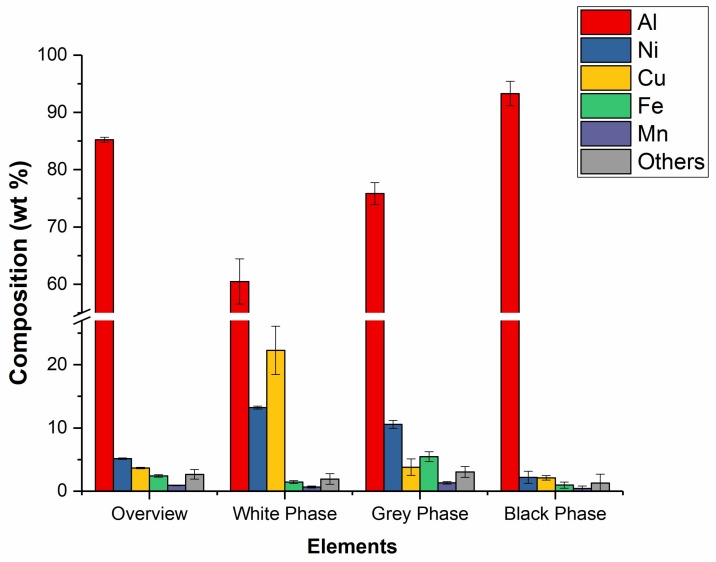
Weight percentage of compositions of the overview and 3 phases [28].

The elemental composition of the sample was analyzed by energy dispersive X-ray (EDX) measurement at 5 different locations for the respective phases and the overview taken at 50× magnification. The elemental compositions were charted in Figure 7. Among the 3 phases, the black phase is mainly composed of Al with a weight percentage of over 90%, while the white phase had the lowest weight percentage of Al composition around 60%. The white phase contained relatively higher amount of Ni and Cu, and the grey phase contained relatively higher amount of Ni and Fe, which implies that the white and grey phases should have a higher hardness.

**Figure 8:**
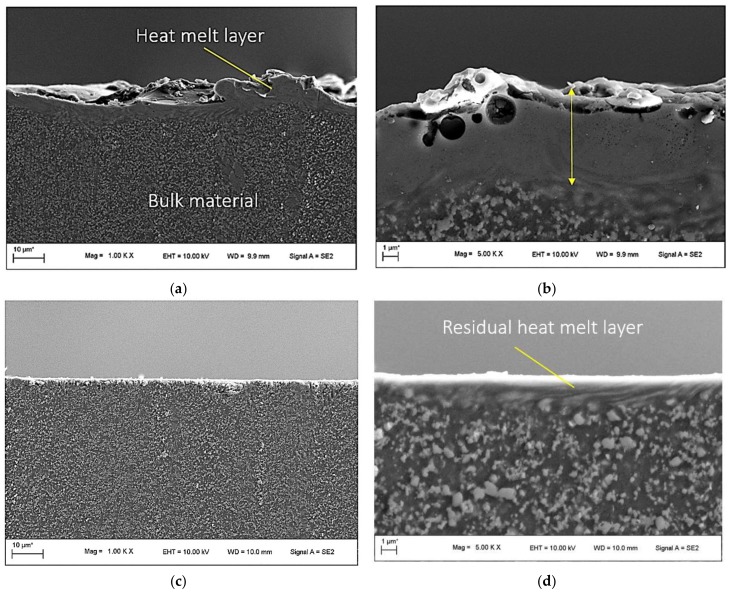

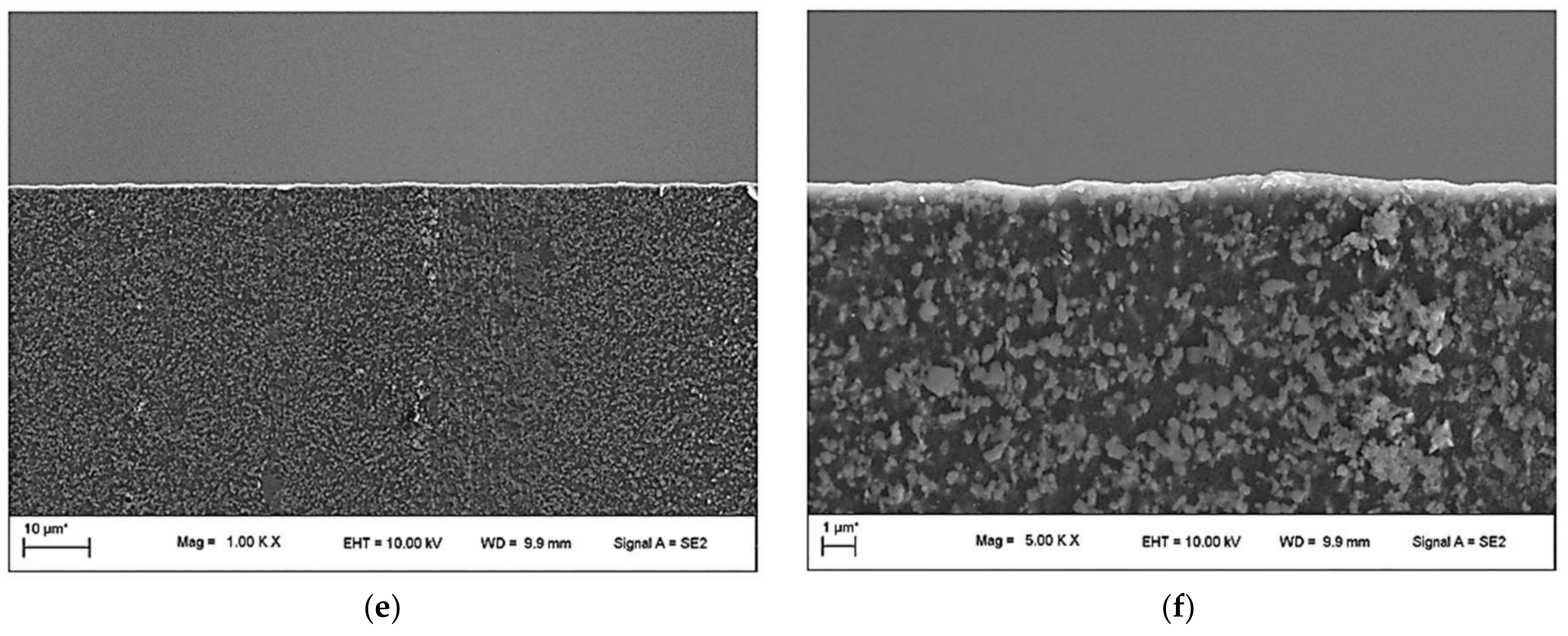
The cross-sectional views of edges before polishing at (**a**) 1k× magnification and (**b**) 5k× magnification, after polishing of sample 3 at (**c**) 1k× magnification and (**d**) 5k× magnification, and after polishing of sample 5 at (**e**) 1k× magnification and (**f**) 5k× magnification.

With the aim of evaluating the influences of wire EDM and MFAF processes on the subsurface of RSA 905, the cross sections of the edges were examined. As shown in Figure 8a,b, after wire EDM, an uneven heat melt layer of a maximum thickness about 10 µm was formed on the sample surface. After MFAF under the condition of Test 3, as the material removal was less than 10 µm, the heat melt layer was not completely removed with a remaining depth of 1–2 µm. Under the condition of Test 5, the layer was completely removed without inducing new damages such as deformed layer or cracks.

### 3.3. Surface Residual Stress and Microhardness Measurements

**Figure 9:**
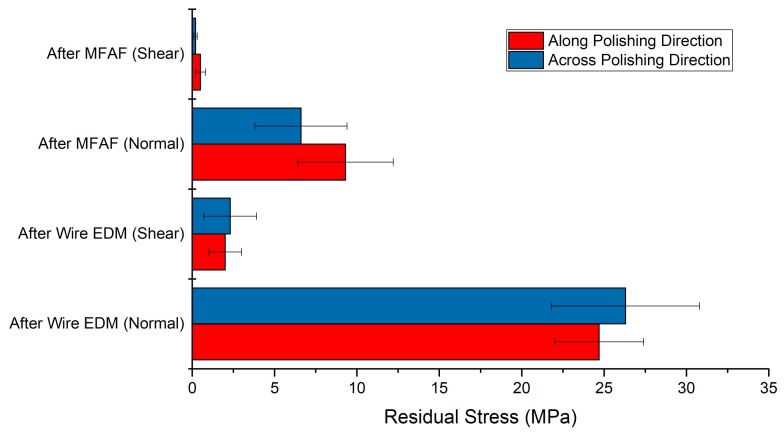
Results of residual stress measurement by XRD.

To evaluate the surface and subsurface quality from another aspect, the respective surface residual stresses after wire EDM and after polishing using test sample 5 were measured using an X-ray Diffractometer (XRD). The penetration depth of X-ray in sample surface was about 80 µm. As shown in Figure 9, it is found that, both normal (perpendicular to the sample surface) and shear (parallel to the sample surface) tensile stresses were released after MFAF process. The normal stresses were reduced by over 50%, and the shear stress became almost zero. Although the values were not absolute, according to the results of cross-sectional observation, it can be concluded that the surface residual stresses were almost fully released.

**Figure 10:**
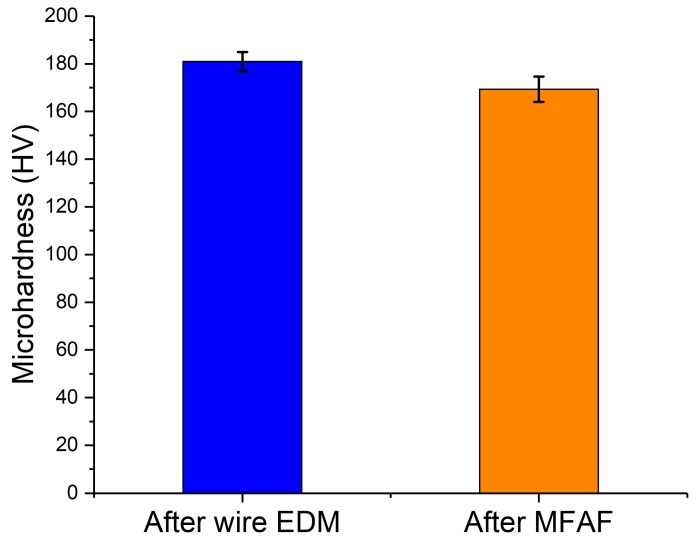
Results of microhardness test.

The surface microhardness before and after polishing was also tested using a micro-Vickers hardness tester at 5 different locations. The applied load was 100 gf. As shown in Figure 10, the results indicated that the heat melt layer hardened the surface, and the microhardness was slightly reduced after MFAF from about 180 HV to 170 HV. The changes in microhardness are consistent with the results of residual stress release.

### 3.4. Tribology Tests

**Figure 11:**
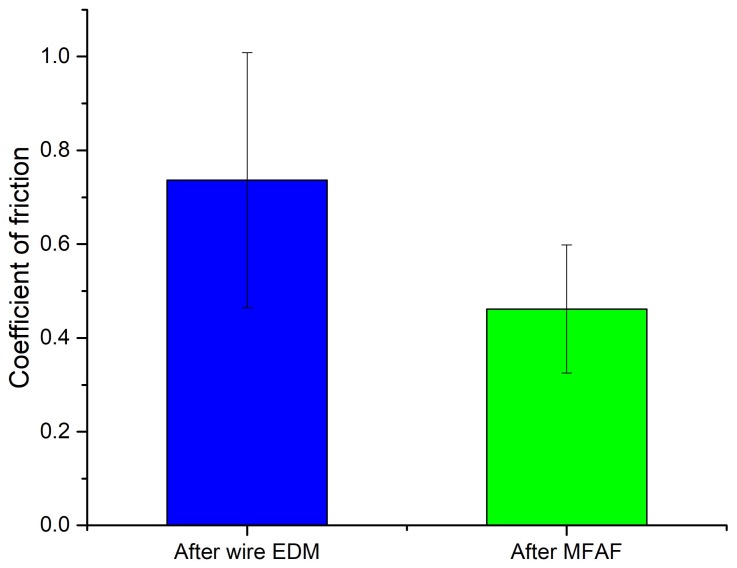
Results of tribology testing.

To evaluate the effect of processes on friction and wear characteristics of the processed sample surface, tribology testing was conducted on both the wire EDMed surface and polished surface of test sample 5 using a UMT-3MT tribometer. The load was 2 N and the time duration was 10 min. The stainless ball with a diameter of 6 mm was used for the test. As shown in Figure 11, the coefficient of fiction was reduced after the polishing process as the surface became smoother. Through measuring the width of the wear trace using Alicona InfiniteFocus scanning microscope, the wear rate was calculated by Equation (1) [29]:

(1) $$Wear\;rate=\frac{B}{Ld}\left[\frac{\pi r^{2}}{180}sin^{-1}\left(\frac{b}{2r}\right)-\frac{b}{2}\sqrt{r^{2}-\frac{b^{2}}{4}}\right]\left[{\mathrm{mm}}^{3}/\mathrm{Nm}\right]$$

where *B* is the trace of friction, *r* is the semi diameter of the chromium steel ball (mm), *b* is the width of the wear trace (mm), *L* is the load (N) and *d* is the sliding distance (m). After calculation, the wear rate was reduced by 40%, from 11.8 × 10^−7^ mm^3^/Nm to 7.2 × 10^−7^ mm^3^/Nm after polishing which indicates that the polished surface provides a lower coefficient of fiction and better wear resistance.

## 4. Conclusions

In this paper, the material removal characteristics of rapidly solidified aluminum RSA 905 by magnetic field-assisted finishing (MFAF) was experimentally studied. The effect of abrasive and polishing speed conditions on material removal and surface roughness was quantitatively investigated. The surface and subsurface quality was evaluated by optical microscope and scanning electron microscope (SEM) observations, residual stress measurement, surface microhardness and tribology test. Based on the results obtained from this research, some conclusions can be drawn as follows:

- At the polishing speed of 400 rpm, the SiC-based magnetic abrasives produced a higher material removal than the Al_2_O_3_-based magnetic abrasives, and large particles contributed to a higher material removal. However, higher polishing speed did not contribute to a higher material removal for large particles since large particles became susceptible to being scattered and dropped out from the abrasives.
- The results of ANOVA analysis showed that at the 0.05 level abrasive type is the critical factor for the developed MFAF process in terms of surface roughness Ra and all the parameters are dominant for the entire MFAF process regarding material removal.
- According to the results of cross sectional observation, surface residual stress and microhardness measurements, the subsurface damage caused by wire EDM was almost completely removed by the MFAF process without inducing new subsurface damage.
- The polished surface showed a better tribological performance than the wire EDMed surface represented by the lower coefficient of fiction and wear rate.

Therefore, a smooth surface without surface and subsurface damage can be obtained by the developed MFAF process in this paper. The experimental results well demonstrate the feasibility of the MFAF processes for finishing the rapidly solidified aluminum alloy RSA 905 and that high machining efficiency with good surface quality is achievable for practical application with the optimal process parameters.

## Figures and Tables

**Table 1:** Experimental conditions.

Equipment	Dual Magnetic Roller Tool
Workpiece material	RSA 905
Initial roughness *R_a_*	>3.0 µm
Tool rotation speed	400 rpm, 800 rpm
Robot arm feed speed	288 mm/min
Gap	1.5 mm
Abrasive type	Al_2_O_3_, SiC
Abrasive size	5 µm, 12 µm

**Table 2:** Design of experiments in MFAF.

Test No.	Abrasive Type	Abrasive Size (µm)	Polishing Speed (rpm)
1	Al_2_O_3_	5	400
2	SiC	5	400
3	Al_2_O_3_	12	400
4	SiC	12	400
5	Al_2_O_3_	5	800
6	SiC	5	800
7	Al_2_O_3_	12	800
8	SiC	12	800

**Table 3:** 3-way ANOVA of surface roughness Ra.

	DOF	SS	MS	*F* Value	*P* Value
Abrasive Type	1	0.01153	0.01153	16.63816	8.74249 × 10^−4^
Abrasive Size	1	3.375 × 10^−4^	3.375 × 10^−4^	0.4871	0.49524
Speed	1	0.00129	0.00129	1.86277	0.19119
Abrasive Type * Abrasive Size	1	0.0805	0.0805	116.18859	9.57754 × 10^−9^
Abrasive Type * Speed	1	0.00101	0.00101	1.46347	0.24395
Abrasive Size * Speed	1	0.00224	0.00224	3.23676	0.09089
Abrasive Type * Abrasive Size * Speed	1	0.041	0.041	59.17758	9.18327 × 10^−7^
Model	7	0.13792	0.0197	28.43635	7.02693 × 10^−8^
Error	16	0.01109	6.92875 × 10^−4^	0	0
Corrected Total	23	0.14901	0	0	0

(DOF: degrees of freedom; SS: sum of squares; MS: mean sum of squares; * means the interaction between factors).

**Table 4:** Significant factors for different parameters at the 0.05 level.

	Ra	MR/50	MR/100	MR/150	MR/200
Abrasive Type	Y	Y	Y	Y	Y
Abrasive Size	N	Y	Y	Y	Y
Speed	N	Y	Y	Y	Y
Abrasive Type * Abrasive Size	Y	Y	Y	Y	Y
Abrasive Type * Speed	N	Y	Y	Y	Y
Abrasive Size * Speed	N	Y	Y	Y	Y
Abrasive Type * Abrasive Size * Speed	Y	Y	Y	Y	Y

(Ra: surface roughness; MR/50/100/150/200: material removal after a 50/100/150/200-minute polishing; * means the interaction between factors).
